# Structure of a Novel Shoulder-to-Shoulder p24 Dimer in Complex with the Broad-Spectrum Antibody A10F9 and Its Implication in Capsid Assembly

**DOI:** 10.1371/journal.pone.0061314

**Published:** 2013-04-19

**Authors:** Ying Gu, Fang Cao, Lei Wang, Wangheng Hou, Jun Zhang, Choy-leong Hew, Shaowei Li, Y. Adam Yuan, Ningshao Xia

**Affiliations:** 1 National Institute of Diagnostics and Vaccine Development in Infectious Disease, School of Life Sciences, Xiamen University, Xiamen, China; 2 Department of Biological Sciences and Centre for Bioimaging Sciences, National University of Singapore, Singapore, Singapore; National Institute for Viral Disease Control and Prevention, CDC, China

## Abstract

Mature HIV-1 viral particles assemble as a fullerene configuration comprising p24 capsid hexamers, pentamers and dimers. In this paper, we report the X-ray crystal structures of the p24 protein from natural HIV-1 strain (BMJ4) in complex with Fab A10F9, which recognizes a conserved epitope in the C-terminal domain of the BMJ4 p24 protein. Our structures reveal a novel shoulder-to-shoulder p24 dimerization mode that is mediated by an S-S bridge at C177. Consistent with these structures, the shoulder-to-shoulder dimer that was obtained from the BMJ4 strain was also observed in p24 proteins from other strains by the introduction of a cysteine residue at position 177. The potential biological significance was further validated by the introduction of a C177A mutation in the BMJ4 strain, which then displays a low infectivity. Our data suggest that this novel shoulder-to-shoulder dimer interface trapped by this unique S-S bridge could represent a physiologically relevant mode of HIV-1 capsid assembly during virus maturation, although Cys residue itself may not be critical for HIV-I replication.

## Introduction

The mature HIV-1 capsid is a fullerene cone comprising approximately 250 hexamers and 12 pentamers of the viral CA protein (p24), with seven pentamers located at the broad end and five pentamers located at the narrow end [1]–[4]. The assembly of the adjacent hexamers is mediated by the dimerization interface, which is formed by the p24 C-terminal domain (CTD) [5]–[9]. Mature HIV-1 forms hexagonal arrays of hexameric rings [10], which are composed of the p24 N-terminal domain (NTD), with each ring connected to its six nearest neighbors via the CTD of p24 through a CTD-CTD dimerization interface [11]–[13]. The flexible nature of the p24 NTD and CTD affects the relative positions of the NTD and CTD within the hexagonal arrays of hexameric rings, which effectively changes the tilt of the dimerization interface relative to the hexamer plane [10].

The HIV-1 p24 protein has been proposed to form a dimer in solution under certain conditions that promote capsid assembly [4]. Both full-length p24 and p24-CTD contain a rapidly equilibrating mixture of monomers and dimers. Notably, the dimeric affinity of p24-CTD displays 2 fold stronger than that of full-length [14]. HIV-1 p24 CTD domains from different constructs have been crystallized into several distinct dimerization interfaces: the CTD domain with full affinity displays a side-by-side conformation that is primarily stabilized by helix 9 (pdb code 1a43) [15], the CTD domain with a shorter construct displays a similar side-by-side dimer with a tilted dyad axis (2a8o) [6], a single amino acid residue deletion introduced in the helix 8/9 linker promotes a “domain-swapping” dimerization mode (2ont) [14], and an HIV-1 inhibitor bound to p24 CTD induces an “inert” CTD-CTD dimer that is incapable of extending the capsid lattice [16]. A recent pseudo-molecular hexagonal lattice structure of the full-length HIV-1 p24 protein, which was derived from a 9 Å resolution electron cryocrystallography map, strongly suggests that the mature HIV-1 capsid lattice is mediated by intermolecular side-to-side CTD-CTD dimerization via helix 9, although the dyad axis is different from the one observed in the side-to-side dimer structure determined from the isolated HIV-1 p24 CTD [6], [13].

Crystallization of the full-length HIV-1 p24 protein is a difficult task because of the intrinsic flexibility of the linker region between the NTD and CTD, the weak interactions within hexamers and the tendency to form capsid-like particles [4], [11], [12]. To overcome this technical difficulty, many approaches have been employed, including disulfide cross-linking by Cys mutations and dimerization promotion by deletion mutations. These efforts have yielded CA hexamer, CA pentamer and “domain-swapping” dimer structures [14]. Although these structures have provided crucial structural information on the process leading to HIV-1 capsid assembly, the mutant structures may not represent the authentic oligomer status of the HIV-1 p24 protein in the native environment.

To determine the structure of the full-length HIV-1 capsid p24 dimer with high dimerization affinity and without the introduction of mutations, we systematically expressed a dozen full-length p24 capsid proteins from different HIV-1 strains. We found that p24 from the BMJ4 strain forms a dimer in solution and in viruses that bud from infected cells. In this paper, we describe crystal structures of the HIV-1 capsid p24 dimer from the BMJ4 strain in complex with the Fab fragment of the broad-spectrum antibody A10F9 with different space groups. Our structures of these complexes demonstrate that A10F9 recognizes a continuous epitope located at α-helix 10 (residues 196 to residues 207) in the C-terminal domain of the BMJ4 p24 capsid protein. To our surprise, the BMJ4 p24 capsid protein formed a novel shoulder-to-shoulder dimer interface, which was cross-linked by an S-S bridge that is contributed by Cys177 in both molecules. This novel shoulder-to-shoulder dimer interface was validated by mutating the cysteine at position 177 of p24 proteins from other HIV strains. Consistent with the structural observations, the BMJ4 p24 C177A mutant remained as monomers in solution, and the BMJ4 HIV-1 strain harboring a C177A mutation displayed a low infectivity. Our data suggest that the shoulder-to-shoulder dimer interface mediated by residue 177 could represent a physiologically relevant mode of HIV-1 capsid assembly during virus maturation, although Cys residue itself may not be critical for HIV-1 replication.

## Results

### p24 Dimer Screening and Complex Formation

**Figure 1:**
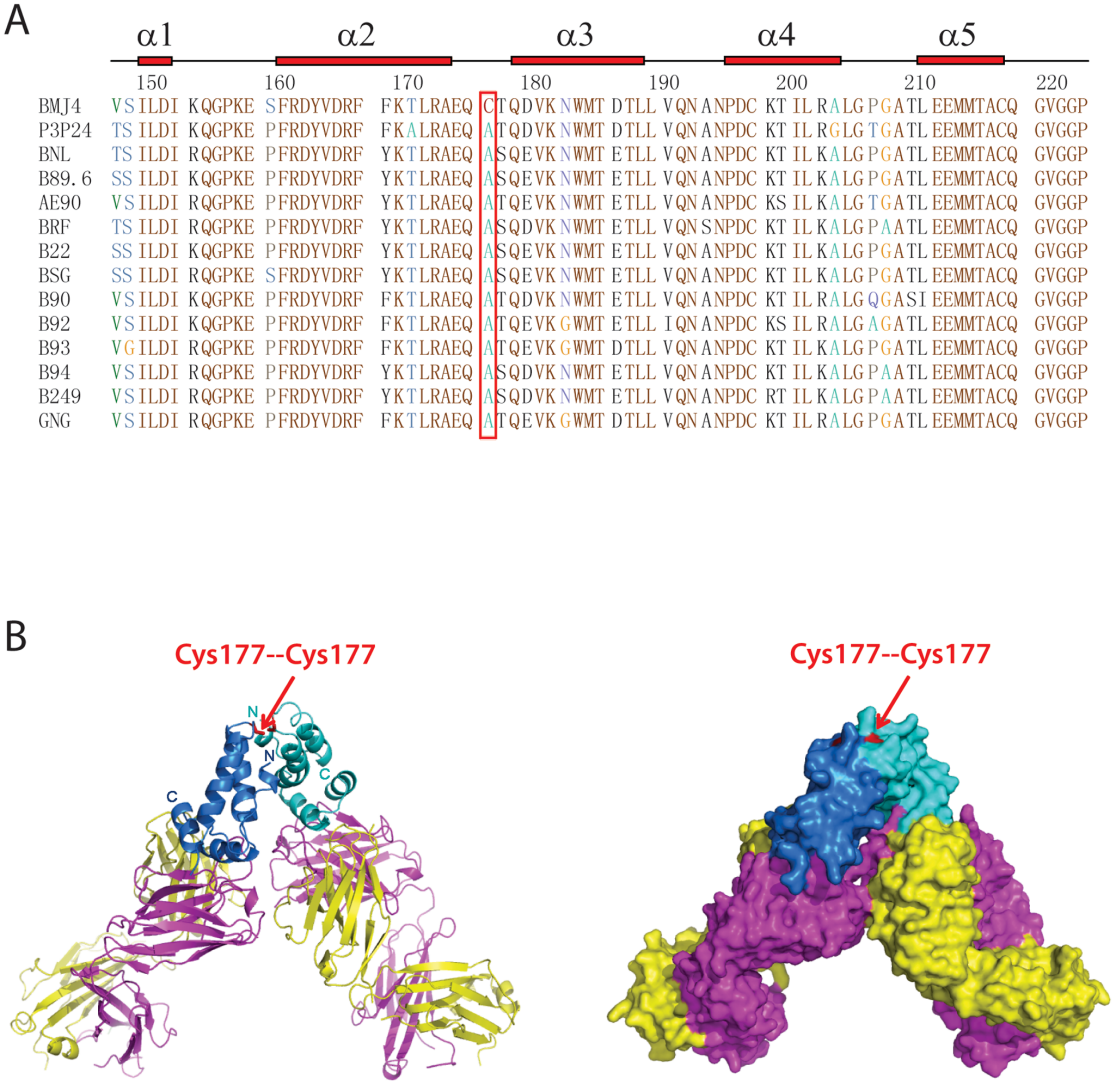
Domain architecture, sequence alignment and overall structure of the HIV-1 p24 capsid protein. (A) Sequence alignment and secondary structure of the HIV-1 p24 capsid protein C-terminal domain (CTD). The aligned sequences are in the following order: BMJ4, P3P24, BNL, B89.6, AE90, BRF, B22, BSG, B90, B92, B93, B94, B249 and GNG. The p24 secondary structure diagram is shown on the top. The α-helices are indicated as bricks. The critical 177 residue is boxed in red. (B) Ribbon (left panel) and surface (right panel) representation of the p24 CTD structure in complex with the A10F9 Fab fragment. The p24 CTDs are in blue and cyan; the Fab light chains are in yellow; and the Fab heavy chains are in magenta. The critical Cys177 residues are labeled in red.

To identify native HIV-1 p24 capsid proteins capable of forming dimers in solution, we systematically screened and expressed a dozen p24 proteins from different HIV-1 strains. Among these, p24 capsid proteins from the BMJ4 strain displayed a stable dimer in solution, and p24 capsid proteins from other HIV-1 strains displayed the monomeric form in solution. Sequence comparison analysis revealed a unique Cys at position 177 of the BMJ4 p24 capsid protein, suggesting that Cys177 could form a disulfide bond to create a stable dimer (Fig. 1A).

To solve the crystal structure of the BMJ4 p24 capsid protein, we screened many p24 monoclonal antibodies that are available in our laboratory. Among these antibodies, the broad spectrum p24 antibody A10F9 displays high binding affinity, specifically targets the BMJ4 p24 CTD domain and demonstrates high binding affinity to live BMJ4 HIV-1 viruses budding from infected cells (Fig. S1). We then prepared and purified the Fab fragment of the A10F9 antibody and made a pure, stable p24 dimer in complex with the A10F9 Fab fragment by using a gel filtration purification approach (Fig. S2).

### Overall Structure of the Complex

Next, we successfully obtained several good crystals of the BMJ4 p24 protein in complex with the A10F9 fragment, after screening more than 100 crystals. These crystals diffract up to 3.2 Å, 3.2 Å and 3.7 Å resolution, which belong to the space groups P2_1_2_1_2_1_, P2_1_ and C222_1_, respectively. The structure of the complex was determined by molecular replacement using the Fab 13B5 fragment (PDBID: 1E6J) as the search model, and the crystallographic statistics are listed in Table 1. The structures of the complexes determined from the three different space groups revealed the same p24 and Fab structures; therefore, only the structure determined from the P2_1_2_1_2_1_ space group at 3.2 Å was used for further structural analysis.

**Table 1:** Data collection, phasing and refinement statistics for BMJ4 p24 capsid-A10F9 Fab complex.

*Data collection*	
Cell parameters (Å)	a = 83.11, b = 124.06, c = 150.83
Space group	P2_1_2_1_2_1_
Resolution range (Å)	50.0-3.2 (3.26–3.20)
Wavelength (Å)	0.9793
Redundancy	14.0 (14.0)
Completeness (%)	99.9 (100)
Overall (I/σI)	25.2 (6.8)
Rsym (%)^a^	12.7 (44.8)
***Refinement***	
Resolution range (Å)	50–3.2
Number of Reflections	24,996
R _work_ (%)	21.1
R _free_ (%)	29.2
No. atoms Protein	7,806
B-factors (Å^2^) Protein	71.1
RMSD bond lengths (Å)	0.015
RMSD bond angles (°)	1.7
***Ramachandran Plot***	
Most favored region (%)	83.2
Disallowed regions (%)	0.5

a Values for the highest-resolution shell are in parentheses.

**Figure 2:**
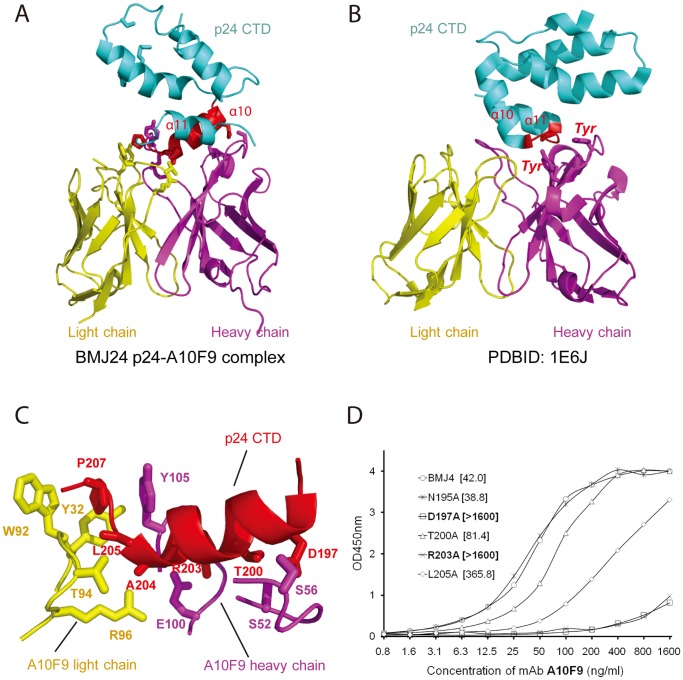
Detailed structural features of p24 CTD recognition by Fab. (A) Ribbon representation of an overall view of p24 CTD recognition by the A10F9 Fab fragment. The p24 CTD is in blue, the Fab light chain is in yellow, and the Fab heavy chain is in magenta. The p24 α-helix (α10) recognized by the Fab fragment is in red. The residues involved in protein-protein interactions are shown in stick mode. (B) Ribbon representation of an overall view of p24 CTD recognition by Fab13B5 (PDBID: 1E6J). The p24 CTD is in blue, the Fab light chain is in yellow, and the Fab heavy chain is in magenta. The short loop between α10 and α11 of p24, which is recognized by Fab, is in red. The Tyr residues that are involved in protein-protein interactions are shown in stick mode. (C) Ribbon representation of a detailed view of p24 CTD recognition by the A10F9 Fab fragment. The residues involved in protein-protein interactions are indicated and shown in stick mode. (D) EC_50_ calculation of BMJ4 p24 and its mutants after precipitation with the A10F9 mAb. The OD_450 nm_ vs. the A10F9 ELISA concentration curves were fitted to the half point of the effective concentration (EC_50_), and the values are shown in brackets.

**Figure 3:**
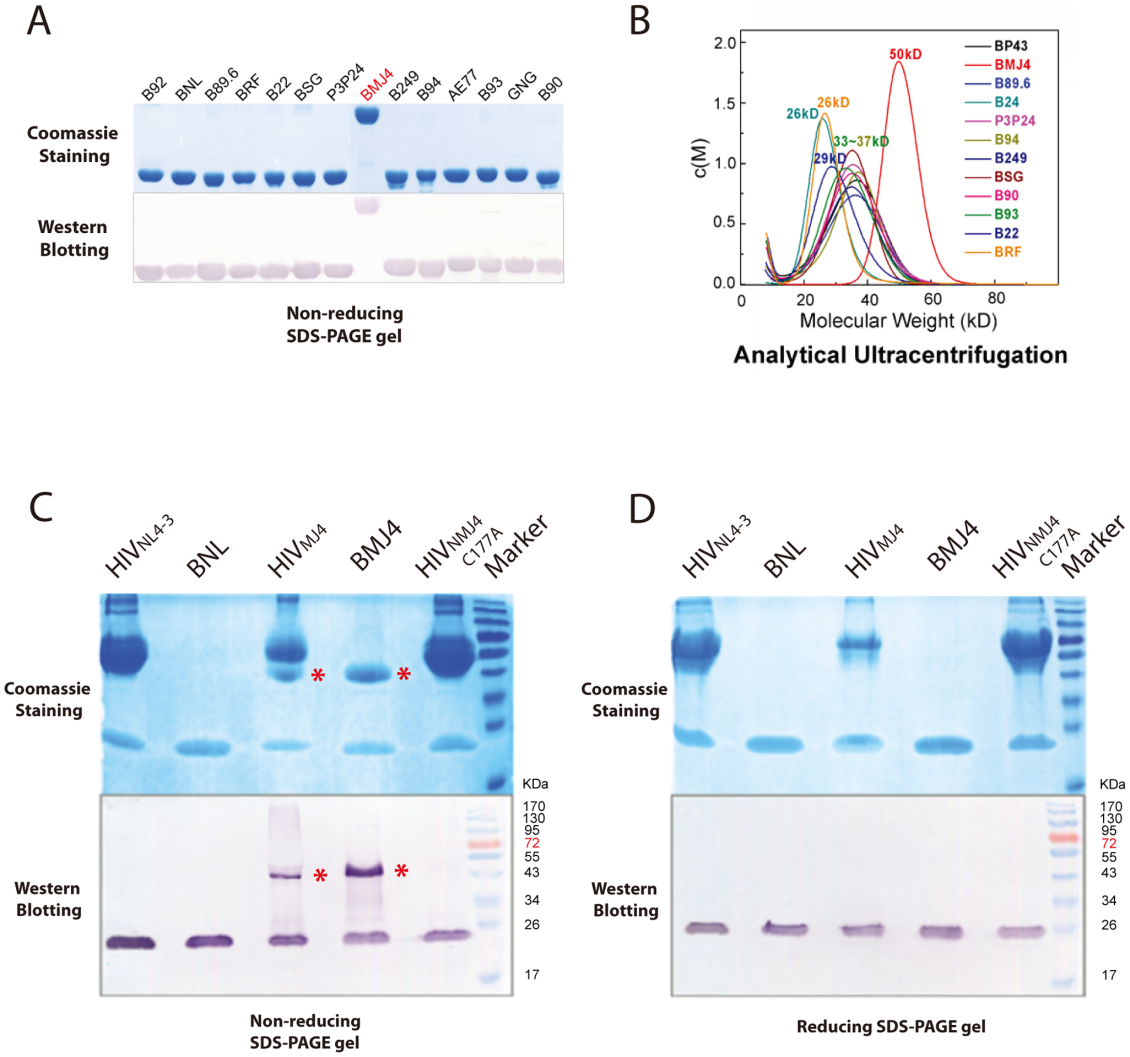
The BMJ4 p24 protein forms a dimer in solution and in budding viruses. (A) Recombinant p24 proteins encoded by different HIV CA genes representing various subtypes were subjected to non-reducing SDS-PAGE. Only BMJ4 p24 displays a dimeric form, as determined by non-reducing SDS-PAGE. (B) The c(M) profiles resulting from sedimentation velocity (SV) were used to determine the apparent MW of the various p24 proteins in solution. The BMJ4 p24 protein displays a dimeric form, which is consistent with gel electrophoresis analysis. (C) p24 proteins extracted from an HIV lysate that was infected by infectious clones, including NL 4–3, MJ4 and the MJ4 C177A mutant, were subjected to non-reducing SDS-PAGE. Dimerization associated with the disulfide bond contributed by residue 177 was validated in a native HIV virion. The p24 dimers are indicated by red stars. (D) p24 proteins extracted from HIV lysates infected by infectious clones, including NL 4–3, MJ4 and the MJ4 C177A mutant, were subjected to reducing SDS-PAGE. The p24 dimers formed by disulfide bonds were dissociated under reducing conditions.

In our structure that was crystallized in the P2_1_2_1_2_1_ space group, there are two copies of noncrystallographically (NCS) related Fab fragments bound to one p24 capsid dimer in one asymmetric unit (Fig. 1B). In our structure, the A10F9 Fab fragment, which was well resolved in our electron density map, displays a canonical sandwich Ig fold. In our structure, the p24 CTD is well resolved, as determined by an excellent density map, whereas the p24 NTD is not resolvable because of a poor density map. Similar to published p24 structures, the p24 CTD monomer maintains the global fold comprising a short 3_10_ helix followed by an extended β-strand and four α-helices. As expected, the interaction surface between p24 and the A10F9 fragment is located adjacent to a continuous epitope in α-helix 10 of the p24 CTD (Fig. 1B and Fig. 2A). Surprisingly, the BMJ4 p24 CTD dimer displays a novel shoulder-to-shoulder dimer interface, which is mediated by the covalent bond contributed from Cys177. Unlike the published p24 CTD dimer, few hydrogen bonds or salt bridges are involved in BMJ4 p24 capsid protein dimerization (Figs. 1B, 3A).

### Interactions between p24 and the A10F9 Fab Fragment

In our structure of the complex, the A10F9 Fab fragment recognizes a continuous epitope that resides in α-helix 10 of the p24 CTD via the hydrophobic loops within the variable regions of A10F9. The interaction interface buries a total of 1,140 Å^2^ of the surface area. The close interactions between p24 and A10F9 are grouped into a collection of aromatic π-stacks at the end of α-helix 10 and numerous hydrogen bonds along one face of α-helix 10. Both the heavy and light chains of A10F9 contribute to p24 CTD recognition (Figs. 2A, 2C). Notably, p24 Pro207 is buried within an aromatic cage formed by Trp92 and Tyr32 from the light chain and Tyr105 from the heavy chain (Fig. 2C). Because Pro207 is located at the end of α-helix 10, making a sharp turn leading to α-helix 11, the extensive π–π interactions contributed by the aromatic cage may serve a critical role by anchoring the p24 CTD. The molecular interactions between the p24 CTD and the A10F9 Fab fragment are further supplemented through intermolecular hydrogen bonds between the main chains of Leu205 and Ala204 from p24 and the Thr94 and Arg96 side chains of the Fab light chain and the hydrogen bonds between the p24 Arg203, Thr200, and Asp197 side chains and the Fab heavy chain Ser52, Ser56, and Glu100 side chains (Fig. 2C).

These structural observations are consistent with our in vitro binding assay of numerous p24 mutants using the A10F9 antibody. The introduction of an Ala mutation at Arg203 disrupts the hydrogen bonds formed between Glu100 of A10F9 and Arg203 of p24, the introduction of an Ala mutation at T200 disrupts the hydrogen bonds formed between Ser52 of A10F9 and Thr200 of p24, and the introduction of an Ala mutation at Asp197 disrupts the hydrogen bonds formed between Ser56 of A10F9 and Asp197 of p24, which significantly reduces the binding affinity (quantified as EC_50_) between p24 and A10F9 (Fig. 2D). In contrast, the introduction of an Ala mutation at a site that is distant from the binding surface, such as N195A, has no impact on the binding affinity of A10F9 for p24 (Fig. 2D). Notably, the introduction of an Ala mutation at Leu205 significantly reduces the binding affinity, suggesting that the hydrophobic interaction between Leu205 of p24 and Tyr32 of A10F9 is crucial for binding (Figs. 2C, D).

### Shoulder-to-shoulder HIV-1 p24 Dimer

Our p24-A10F9 complex structure demonstrates that the BMJ4 p24 capsid protein forms a dimer in the crystal structure (Fig. 1B), which is supported by the observation that the BMJ4 p24 capsid protein forms a dimer in solution, and this result was validated by native gel and analytical ultracentrifugation experiments (Figs. 3A, B). By contrast, the p24 molecules from other HIV-1 isolates contain a rapidly equilibrating mixture of monomers and dimers in solution at our condition (Fig. 3B). To further investigate whether BMJ4 p24 capsid protein dimerization occurs *in vivo*, the BMJ4 p24 capsid protein was isolated from HIV-1 viruses that bud from infected cells. Strikingly, the BMJ4 p24 capsid protein forms a dimer even in budding HIV-1 viruses, which suggests that the BMJ4 p24 dimerization that we observed in solution might be physiologically relevant (Figs. 3C, D). Consistent with this idea, the BMJ4 p24 dimer structure that was determined by three different space groups reveals an identical novel shoulder-to-shoulder dimer interface. Within our structure of the complex, the p24 CTD dimer portion (residues 149–221) was traced without ambiguity, whereas the p24 NTD portion was impossible to trace because of the low-quality density map. Nevertheless, our structure demonstrates that the p24 dimer interface is mediated by the p24 CTD, whereas the p24 NTD is not involved in dimerization.

**Figure 4:**
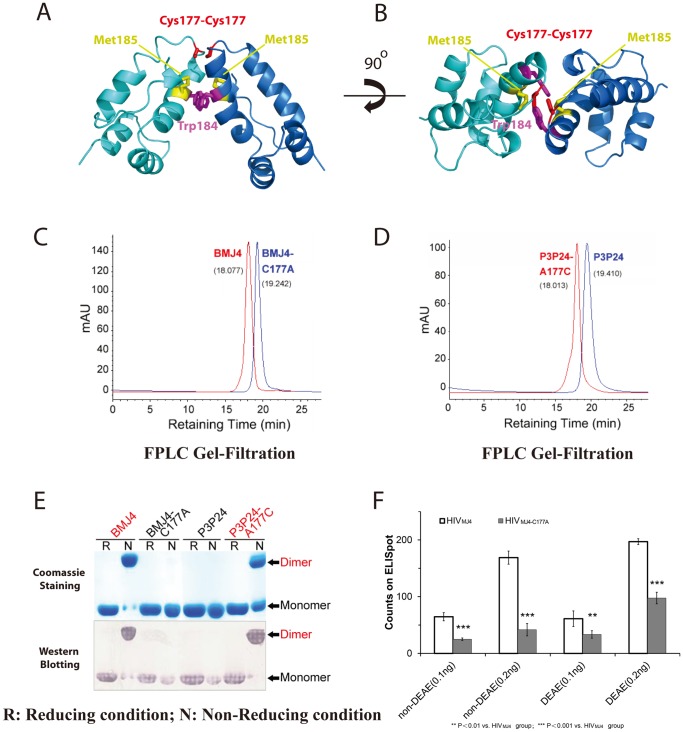
The HIV-1 p24 capsid protein dimer interface is mediated by residue 177. (A) Ribbon representation of the BMJ4 p24 CTD dimer. BMJ4 p24 CTD dimers are in blue and cyan. The critical Cys177 residues are shown in stick mode in red, the conserved Trp184 residues are shown in stick mode in magenta, and the Met 185 residues are shown in stick mode in yellow. (B) A 90° rotation of (A) along the X-axis. (C) Analytic gel filtration is used to determine the apparent MW of BMJ4 p24 and its C177A mutant in solution. Disulfide bond disruption disrupts the stable dimer formation in solution, which is consistent with the gel electrophoresis analysis. (D) Analytic gel filtration is used to determine the apparent MW of P3 p24 and its A177C mutant in solution. The introduction of the disulfide bond triggers stable dimer formation in solution, which is consistent with gel electrophoresis analysis. (E) Validation of the critical role of Cys177 in stable dimer formation in solution. Recombinant p24 proteins encoded by BMJ4 and P3P24 were first treated under non-reducing (N) and reducing conditions (R) and subjected to SDS-PAGE. Monomers and dimers are indicated by the arrows. Disruption of the disulfide bond through the C177A mutation in the BMJ4 p24 protein disrupts stable dimer formation in solution, and the introduction of a disulfide bond through the A177C mutation in the P3 p24 protein triggers stable dimer formation in solution. (F) Infection activity of the HIV MJ4 strain and its p24 C177A mutant strain. The cells were infected with two HIV titers (quantified by p24-based ELISA) in the presence or absence of 15 µg/ml DEAE/dextran. The average and standard error of five replicates of ELISpot counts are shown. P-value analysis was applied to statistic evaluation for infectivity difference.

Detailed structural analysis demonstrates that the p24 CTD dimer interface is mediated by a single disulfide bond, which is provided by Cys177 in two monomers (Fig. 4A). Similar to previously reported p24 CTD dimers, BMJ4 p24 is dimerized by the parallel packing of α-helix 9 across the dimer interface. However, few hydrogen bonds and/or salt bridges are involved in dimerization in our BMJ4 p24 dimer structure (Fig. 4B). The reported π–π interaction that is contributed by the conserved Trp184, which is found along the dimer interface, is not observed in our BMJ4 p24 dimer structure. In our structure, the distance between the Trp184 residues of two monomers is 7 Å, and they are not π-stacked on each other (Fig. 4B). In our BMJ4 p24 dimer structure, α-helix 9 is well folded to form a standard α-helix, whereas Cys177 is located in the loop linking α-helix 9 and α-helix 10, which is ∼4 Å away from Trp184. Therefore, our shoulder-to-shoulder dimer displays a novel dimer interface compared with previously published p24 dimer interfaces (Fig. 4A, B).

The loose interaction between p24 monomers revealed by our BMJ4 p24 dimer structure is consistent with the low dimerization affinities displayed by both the p24 and p24 CTD dimers in solution. In contrast, the large solvent-accessible surface areas observed in other p24 dimer structures, such as the domain-swapping dimer interface, are probably due to dimer interface rearrangement that is driven by packing force.

We speculate that the p24 dimer interface could only be trapped by HIV-1 capsid assembly force due to the weak interactions between p24 monomers. The disulfide bond contributed by BMJ4 p24 Cys177 provides a unique opportunity to lock the p24 dimer interface before HIV-1 capsid assembly. Indeed, a BMJ4 p24 capsid mutant protein bearing the C177A mutation displays a monomeric form in solution (Fig. 4C, 4D) and budding HIV-1 viruses (Fig. 3C).

To investigate whether this shoulder-to-shoulder dimer interface mediated by residue 177 is a universal structural feature of other HIV-1 strains, we introduced a Cys at residue 177 in the p24 capsid protein from the P3P24 strain. As expected, the P3P24 p24 capsid protein formed a dimer in solution after the introduction of a disulfide bond at residue 177 (Figs. 4C, 4E). Therefore, it is conceivable that p24 capsid proteins from other HIV-1 strains dimerize at residue 177, and the introduction of a Cys at position 177 will trigger stable p24 dimer formation in solution.

To further investigate whether the stable dimer interface mediated by C177 is crucial for the HIV-1 BMJ4 strain function *in vivo*, a BMJ4 C177A mutant strain was created to infect human cells in culture to evaluate the infectivity. Strikingly, the introduction of a C177A mutation in the HIV-1 BMJ4 strain not only disrupted BMJ4 p24 capsid dimerization in the budding virus (Fig. 3C) but also compromised the amplification rate and infectivity of the HIV-1 BMJ4 strain in cultured cells (Fig. 4F). Therefore, the novel shoulder-to-shoulder p24 dimer interface mediated by residue 177 that was identified in the BMJ4 p24 dimer could represent a physiologically relevant mode of HIV-1 capsid assembly during virus maturation, although Cys residue itself may not be critical for HIV-I replication.

## Discussion

### Comparison with Other Antibodies Recognizing HIV-1 p24 CTD

Monoclonal antibodies (mAbs) with a broad capacity to neutralize the infectivity of diverse HIV-1 strains are being pursued as protective passive vaccines against HIV-1. The envelope glycoprotein spike (Env) of HIV-1 is one of the primary target for vaccine development, but the HIV-1 capsid has also been considered as a targets for vaccine development [10], [17]–[19]. In the literature, several Fab fragment crystal structures of mAbs raised against HIV-1 p24 have been reported; some Fab fragments specifically recognize the p24 NTD, whereas others specifically recognize the p24 CTD [20]–[22].

We generated several dozen monoclonal antibodies that are directed against recombinant HIV-1 p24 proteins and have high binding affinities, thus providing a monoclonal antibody pool with which to identify the unique characteristics of antibodies specific for p24 proteins from different HIV-1 strains. We have successfully identified that the monoclonal antibody A10F9 displays a broad substrate spectrum and high binding affinity for BMJ4 p24 in vitro and ex vivo (Figure S4). Our protein-protein interaction assay, which was followed by structural mapping, demonstrates that A10F9 specifically recognizes the BMJ4 p24 CTD (Fig. S3). Our detailed structural analysis of the complex structure reveals that the Fab heavy chain Ser52, Ser56 and Glu100 residues and the Fab light chain Thr94 and Arg96 residues form hydrogen bonds with residues at one surface of p24 α10, whereas the Fab light chain Trp92 and Tyr32 residues and Fab heavy chain Tyr105 residue provide an aromatic cage that recognizes the invariable Pro207 residue of p24 (Fig. 2C).

Next, we tested whether these residues involved in p24 CTD recognition in the A10F9 Fab structure are conserved in other monoclonal antibody structures. We performed a structural comparison of our Fab fragment structure with the crystal structure of the Fab13B5 fragment recognizing the C-terminal domain of an intact p24 mutant at 3 Å resolution (PDBID: 1E6J) [20]. Notably, mAb 13B5 recognizes a nearly continuous epitope within a loop (residues 205–213) between α-helix 10 and α-helix 11 in the p24 CTD with a relatively small contact area, which is mostly contributed by heavy chain residues (Fig. 2B) [21]. In contrast, our mAb A10F9 antibody recognizes a continuous epitope along one surface of α-helix 10 (residues 196–207) in the p24 CTD with extensive hydrogen bonds that are contributed from residues in the heavy and light chains (Figs. 2A, 2C). Strikingly, the invariable Pro207 residue seems to be critical for Fab recognition in the 13B5-p24 and A10F9-p24 structures. In the 13B5 structure, the Pro207 residue is recognized by two Tyr residues in the heavy chain by van der Waals interactions (Fig. 2B), whereas in the A10F9 structure, Pro207 is recognized by Trp92 and Tyr32 from the light chains and Tyr105 from the heavy chain through π–π interactions (Figs. 2A, 2C). Interestingly, the Pro207 conformation in p24 was reported to be dramatically changed after Fab binding [20]. Therefore, it is conceivable that Pro207 could be a vaccine development target.

### Comparison with other HIV-1 p24 Dimer Interfaces

The HIV-1 p24 dimer interface determines the relative positions between the two adjacent capsid building blocks and, eventually, capsid assembly. Therefore, high-resolution structures of the p24 dimer should provide important information to understand the molecular insights into HIV-1 capsid assembly. However, many p24 dimer structures with significant deviations are reported in the literature, and no conclusively authentic dimer interface has been validated.

In one case, full-length p24 was reported to form a head-to-tail dimer via extensive intermolecular interactions between the NTD and CTD [20], [23], which, however, is not supported by hydrogen-deuterium exchange and chemical cross-linking experiments [24]. Thus, no functional evidence supports such a dimerization interface.

**Figure 5:**
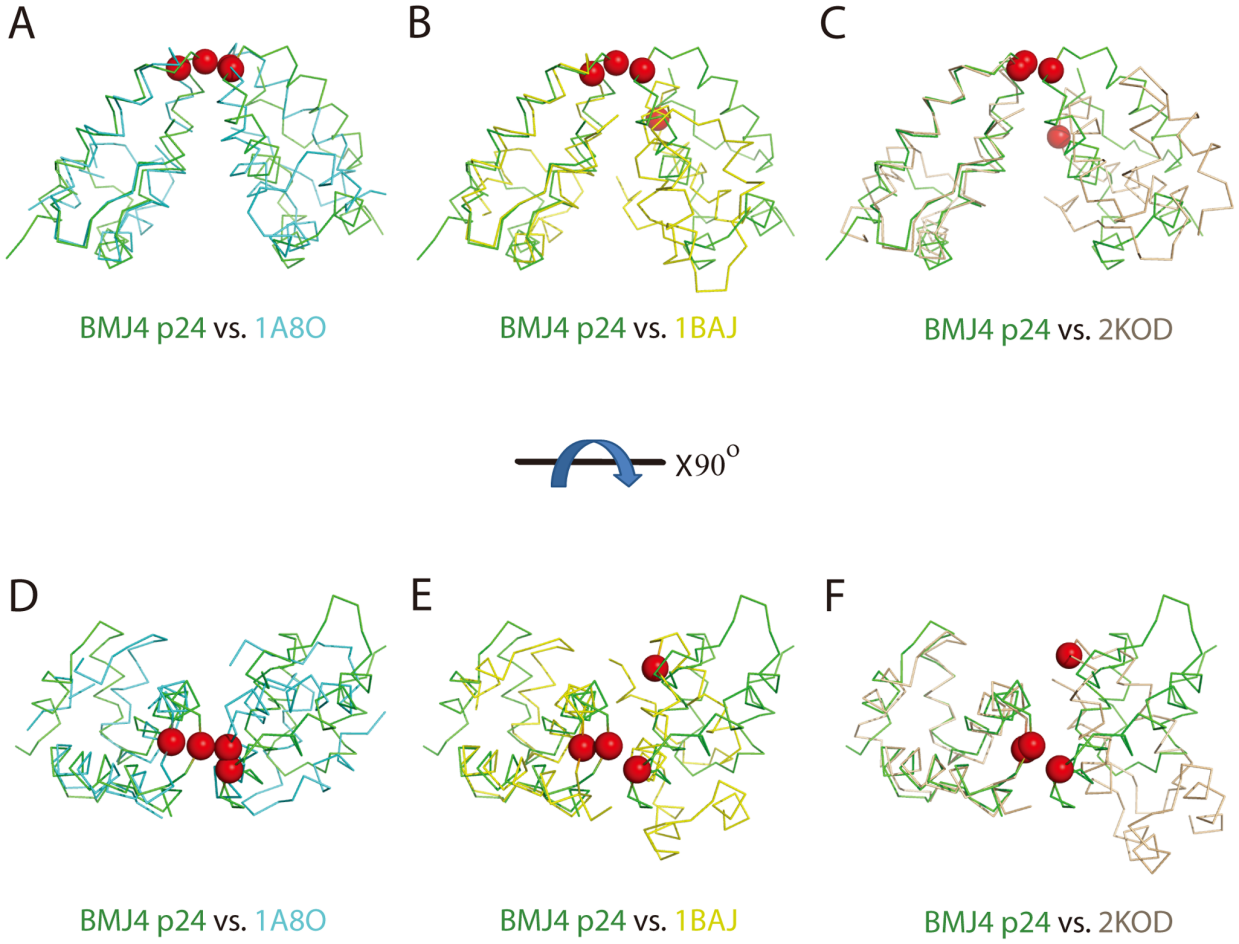
Comparison between the BMJ p24 CTD dimer structure and previously reported CTD dimer structures from X-ray crystallography and NMR. (A, B, C) Backbone atomic superimpositions of the BMJ4 p24 structure (green) and the crystal structures 1A8O (cyan), 1BAJ (yellow) and 2KOD (wheat). Best-fit superimposition is performed for one monomer unit to highlight the difference in subunit orientations in the dimers. The critical 177 residues are marked in the sphere in red. (D,E,F) Rotation of (A, B, C) by 90° around the X-axis.

In the second case, the p24 CTD (residues 151–231) was reported to form a dimer in a head-to-head manner via parallel packing of α-helix 9 across the dimer interface [6] (PDBID: 1A8O) (Fig. 5A). However, constructs that lack the most conserved MHR segment display low dimerization affinity compared with full-length p24 [25]. In contrast, the p24 CTD (residues 146–231), which comprises the MHR segment, displays comparable dimerization affinity as the full-length p24 (PDBID: 1BAJ) (Fig. 5B) [15], [26]. Although both of these p24 CTD constructs are dimerized in a head-to-head manner, the p24 CTD (residues 146–231) construct displays a more packed dimerization interface, with extensive hydrogen bonds and salt bridges (Fig. 5B) [6], [15]. Consistent with the head-to-head CTD dimerization interface that is observed in crystal structures, mutation of Trp184 or Met185 to Ala along this dimer interface abolishes dimerization in solution [6] and blocks capsid assembly in vitro and HIV-1 replication in culture [27].

In the third case, the p24 CTD (residues 144–231) dimer was determined by NMR in solution (Fig. 5C) [13]. Analogous to X-ray structures, the p24 CTD forms a dimer in a head-to-head manner, with extensive hydrophobic interactions between residues at the N-terminus of the CTD and residues residing in α-helix 9. Although the p24 CTD used a similar group of residues, such as Trp184 and Met185, for dimerization, the relative orientation between the two monomers is different. The p24 CTD dimer solved by NMR displays a V-shape-like architecture (Fig. 5C) (PDBID: 2KOD), which is partly supported by the cryo-EM study of the HIV-1 CA tubular structure at 16 Å resolution and an electron density map derived from flattened 2D sheets formed by full-length p24 [11].

In our structure, p24 CTD dimerization is mediated by the same α-helix 9 in a novel shoulder-to-shoulder manner (Figs. 4A, B). Although the p24 CTD monomer structure is almost identical to those reported in crystals and solution (r.m.s.d 1.3 Å∼2.1 Å), our p24 dimer structure displays a significantly different dimer interface compared with those from the reported dimer structures (Fig. 5). Our BMJ4 p24 dimer structure is closer to the p24 CTD dimer structure (residues 151–231) (PDBID: 1A8O), with an approximately 15-degree rotation along the dimerization dyad, which is mediated by α-helix 9 (Fig. 5A, 5D) [6], [15]. Therefore, the previous dimerization residues, including W184 and M185, are further away from their corresponding residues in our BMJ4 p24 dimer structure. Few hydrogen bonds and hydrophobic interactions are observed along this dimerization interface (Fig. 4B). In our structure, Cys177 plays the determining role for dimerization by providing disulfide bonds to link the two monomers together. Notably, Cys177 is located in the loop that links to the beginning of α-helix 9, which is ∼4 Å away from Trp184 (Fig. 4A). Consistent with the importance of the new dimerization interface observed in the BMJ4 p24 dimer, the disruption of this disulfide bond by introduction of the C177A mutation abolishes p24 dimerization in solution and compromises the infection activity of the HIV-1 BMJ4 strain in cultured cells (Fig. 4F).

The comparisons of the three p24-CTD dimers solved by NMR and X-ray crystallography strongly suggest that the p24 CTD is dimerized via α-helix 9 in a CTD-CTD manner. However, the relative orientation of the dimerization interface varies greatly. No evidence has been obtained to support and/or exclude any of the possible models mentioned above. In addition, full-length p24 displays low dimerization affinity in solution, as determined by NMR experiments [13] and our native gels (Fig. 3A). In contrast, the isolated p24 CTD displays elevated dimerization affinity. Therefore, the isolated p24 CTD dimer structures may not represent an authentic dimer interface that is formed by the C-terminal domain of the intact HIV-1 p24 protein. In contrast, the introduction of multiple mutations along the dimerization α-helix and the flexible structural feature of p24 preclude a meaningful interpretation of the p24 dimer interface that is observed in the full-length p24 structures.

Our strategy to identify full-length p24 capsid proteins with elevated dimerization affinities from wild HIV-1 strains should overcome any experimental artifacts introduced by mutations or truncations in p24 molecules. The discovery that the full-length HIV-1 p24 capsid protein from the BMJ4 strain forms a stable dimer in solution and in budding viruses in cultured cells provides the unique opportunity for us to investigate the possible authentic dimer interface of p24 (Fig. 3). The disulfide bond formed by Cys177-Cys177 could reveal a potential physiological dimer interface related to HIV-1 capsid assembly. The existence of such novel shoulder-to-shoulder dimer trapped by S-S bridge is further validated by mutational analysis of isolated p24 capsid proteins and a mutated BMJ4 strain (Figs. 3C, 4F). Moreover, the introduction of a Cys at residue 177 is able to the trigger the p24 capsid protein in other HIV-1 strains to form a stable dimer in solution, strongly suggesting that the involvement of residue 177 in dimer formation could be relevant to HIV-1 capsid assembly (Fig. 4D).

Our new strategy for p24 dimer screening has revealed a novel shoulder-to-shoulder dimer, which is mediated by Cys177. However, we speculate that Cys177 itself may not be critical for HIV-1 replication since many other HIV-1 isolates lack this unique Cys. Therefore, we prefer to argue that many dimer structures could be existed during capsid assembly and this unique Cys177 residue provides us an opportunity to discover the novel shoulder-to-shoulder dimer interface. Although we lack direct evidence demonstrating that the shoulder-to-shoulder dimer interface is present in the mature HIV-1 capsid, we speculate that such a dimer interface may be correlated with one of the dimer interfaces associated with HIV-1 capsid assembly.

## Materials and Methods

### Plasmid and Strain Construction

The HIV-1 capsid CA genes (encoding p24) were generated by commercial DNA synthesis or cloning from isolated HIV strains representing various HIV subtypes, including A, B, B/C, C, D, D/C, F and H subtypes. The genes amplified by PCR were first cloned into the pMD 18-T vector using a TA cloning kit (Takara, Dalian, China) and subsequently subcloned into the non-fusion pTO-T7 vector with an additional N-terminal start Met for expression. The ER2566 *Escherichia coli* strain was used for protein expression. The proteins were named B92 (A), BNL (B), B24 (B), B89.6 (B), BRF (B), B22 (B), BSG (B/C), P3P24 (B/C), BMJ4 (C), B94 (C), B249 (D/C), B93 (F) and B90 (H). Alanine scanning mutagenesis of p24 (BMJ4) was performed using site-directed PCR and confirmed with DNA sequencing. The proviral plasmid pBMJ4 construct carrying the C177A mutation was generated using the QuikChange® Lightning Site-Directed Mutagenesis Kit (Agilent) and confirmed by DNA sequencing. The plasmid carrying the C177A mutation was transfected into 293FT cells for the propagation of HIV-BMJ4-C177A for infectivity evaluation.

### Protein Expression, Purification and Fab Preparation

Protein expression was induced with 0.4 mM isopropyl-β-d-thiogalactopyranoside for 6 h at 25°C. After harvesting, the cells were re-suspended in a buffer containing 50 mM Tris-HCl buffer, pH 7.2, 10 mM EDTA, 300 mM sodium chloride and disrupted by sonication. The supernatant was treated with ammonium sulfate and allowed to precipitate at 4°C, and the p24 protein precipitated at 28% ammonium sulfate saturation. The precipitated p24 protein was re-suspended in 20 mM phosphate buffer, pH 6.0, and further purified by cation-exchange chromatography using CM-5PW (TOSOH, Tokyo, Japan). Monoclonal antibodies were raised from the cross-immunization of p24 proteins from the different HIV subtypes using a standard murine mAb preparation protocol. The broad-spectrum antibodies were identified using the cross-reaction criteria for all of the representative p24 proteins in an ELISA (Figure S4).The A10F9 Fab fragment was obtained by papain digestion of the purified A10F9 antibody followed by anion-exchange chromatography using DEAE-5PW (TOSOH).

### Purification and Crystallization of the p24:A10F9 Complex

The stable p24:A10F9 Fab complex was obtained by incubating the two purified components (referred to Supporting Information) with an excess of A10F9 Fab (molar ratio 1∶1.5) at 37°C for 2 h and further purification with G5000PW (TOSOH) in 20 mM HEPES buffer at pH 7.0. The complex was concentrated to ∼6 mg/ml for crystallization trials. Diffraction-quality crystals were grown by equilibrating 1 µl of the p24:A10F9 complex protein solution and 1 µl of the reservoir solution (0.2 M NaBr, 14% PEG 3350 and 0.2 M NaCl) using the hanging drop vapor diffusion technique at 22°C. These crystals grew to a maximum size of 0.3 mm×0.1 mm×0.05 mm over 2 weeks.

### Structure Determination

Crystals were cryo-protected in the reservoir solution, which was supplemented with 25–30% glycerol, and flash cooled at 100 K. The diffraction data from the p24:A10F9 crystals were collected at the Shanghai Synchrotron Radiation Facility (SSRF) beamline BL17U using a Quantum-315r CCD Area Detector. All of the datasets were processed using the HKL-2000 program package (www.hkl-xray.com). The structures of the BMJ4 p24 dimer in complex with the A10F9 Fab fragment were determined by molecular replacement using Fab 13B5 as the search model (PDBID: 1E6J). The A10F9 Fab fragment part was built and refined first, and the p24 dimer model was subsequently built after several rounds of refinement on the A10F9 Fab model only. The model was built using the O program (http://xray.bmc.uu.se/alwyn) and refined using REFMAC/CCP4 (www.ccp4.ac.uk) with the crystallographic statistics listed in Table 1. Our current complex model includes two A10F9 Fab fragment copies and one p24 CTD dimer (residues 149–221), and the p24 NTD is not included in our final model due to the low quality of its density map.

### SDS-PAGE and Western Blotting (WB)

The analysis of proteins and HIV virions by SDS-PAGE was performed according to the Laemmli method with minor modifications. Polyacrylamide gels containing 12% acrylamide in the separating gel and 5% acrylamide in the stacking gel were used. Protein samples or heat-inactivated HIV samples were mixed with equal volumes of 2x loading buffer (100 mM Tris-HCl, pH 6.8, 200 mM BME, 4% SDS, 0.2% bromophenol blue and 20% glycerol). Sample mixtures were heated at 100°C for 3 min and subsequently loaded in the separating gel. For the non-reducing SDS gel, the buffer contained only 0.1% SDS (no BME), and the sample was not boiled.

For Western blotting experiments, separated proteins were transferred from an SDS gel onto a nitrocellulose membrane. Membranes were soaked in monoclonal antibody (A10F9) diluted 1∶2,000, incubated at room temperature for 1 h, and subsequently washed with 0.2% Tween 20 in phosphate-buffered saline (at pH 7.4). The bound antibody was detected with an alkaline phosphatase-conjugated secondary antibody (DAKO, Glostrup, Denmark), and developed using a mixture of nitro blue tetrazolium and 5-bromo-4-chloro-3-indolyl phosphate.

### Analytical Ultra-centrifugation (AUC)

Sedimentation velocity (SV) was used to determine the molecular weight of p24 in solution. All of the tested samples were diluted to final concentration of 0.9 mg/ml (OD_280 nm_ of ∼1.0 in a 1.2 cm light path in phosphate-buffered saline (pH 7.4). The experiments were conducted at 20°C on a Beckman XL-A analytical ultracentrifuge, equipped with absorbance optics and an An60-Ti rotor. The rotor speed was 45,000 rpm. The sedimentation coefficient was obtained using the c(s) method [28] and then scaled to c(M) profiles for MW calculation using Sedfit software, which was kindly provided by Dr. P. Schuck, National Institutes of Health (http://www.analyticalultracentrifugation.com).

### Gel Filtration HPLC

Purified proteins were passed through a G5000PWxl GFC column that was equilibrated in phosphate-buffered saline (at pH 7.4) using an Agilent1200 HPLC (Agilent, Santa Clara, USA) at a 0.5 ml/min flow rate. The UV absorbance at the 280 nm wavelength was recorded during the elution.

### ELISA

An enzyme-linked immunosorbent assay (ELISA) was performed to evaluate the reactivity of the A10F9 mAb with various p24 proteins and their mutants. Each well of a 96-well microplate was coated with p24 protein (100 ng/well). The solid surface was then incubated with two-fold serial dilutions of the A10F9 mAb at 37°C for 1 h, and the HRP-conjugated GAM secondary antibody was added to the microplate for 30 min at 37°C. Subsequently, 100 µl of tetramethylbenzidine substrate was added, and the plates were incubated for 10 min at 37°C. The reaction was stopped by adding 50 µl 2 M H_2_SO_4_, and the OD was determined at 450 nm and corrected against 620 nm. The EC_50_ (half maximal effective concentration) value of A10F9 binding to p24 was calculated by plotting the dose-response curve as the OD_450 nm_ vs. the mAb concentration. To quantify the HIV p24 protein, a double-antibody sandwich ELISA was performed. The first antibody, anti-p24 mAb 16G12, was coated onto a 96-well microplate. The secondary antibody, anti-p24 mAb2F2, was labeled with HRP. Two-fold serial dilutions of highly purified p24 proteins at 1,400, 700, 350, 175, and 87.5 pg/ml were used for reference standard curve calibration. The p24 concentration in a sample of interest could then be measured by the OD_450 nm_ value determined by the calibration curve.

### Immunofluorescence Confocal Microscopy

To verify that A10F9 recognizes natural p24 in HIV, the pNL4-3 or BMJ4 proviral DNA plasmid was transfected into 293FT cells (10^5^ cells/well, 24-well plate, with glass coverslips preset in the wells) using Lipofectamine 2000 (Invitrogen, Carlsbad, USA). The cells were then cultured for 48 h at 37°C. After washing in PBS, the cells were fixed for 20 min at room temperature in 4% paraformaldehyde (Sigma, St. Louis, USA), permeabilized with 0.3% Triton X-100 (Sigma) for 10 min, washed in PBS, and incubated in goat serum for 1 h. The cells were stained with a primary A10F9 mAb and a secondary goat anti-mouse antibody that was coupled to FITC (Sigma). Nuclei were stained with DAPI (Sigma). Confocal laser scanning microscopy was performed using a Zeiss LSM 780 microscope (Carl Zeiss MicroImaging, Jena, Germany).

### BIAcore Biosensor Analysis

Gold-coated CM-5 sensor chips were coated with a carboxylated dextran polymer matrix in which the GAM-Fc (goat anti-mouse antibody Fc fragment) was amine coupled. The affinity measurement of A10F9 mAb binding to p24 (BMJ4) was initiated by passing HBS over the sensor surface for 100 s at 10 µl/min, and 30 µl of the A10F9 mAb was then injected at the same flow rate. Subsequently, 50 µl of serially diluted p24 was injected. Five concentrations were used for p24 (BMJ4): 2 nM, 4 nM, 10 nM, 20 nM, and 30 nM. Each measurement with the BIAcore3000 biosensor (GE Healthcare, Uppsala, Sweden) was repeated five times, and the mean values were used for affinity constant fitting.

### ELISpot Assay

The infectivity of the HIV_MJ4_ viruses containing Cys177 or Ala177 was evaluated using an ELISpot assay. Briefly, the proviral DNA plasmids pMJ4 and pMJ4-C177A were transfected into 293FT cells using Lipofectamine 2000 (Invitrogen). The propagated HIV in the culture medium was harvested after 72 h and quantified using the above-mentioned double-antibody sandwich ELISA. Two concentrations (3 ng and 6 ng) of p24 from the HIV_MJ4_ and HIV_MJ4-C177A_ virus stocks were added to Tzm-bl cells in a 96-well plate. HIV infections were performed in the presence or absence of 15 µg/ml DEAE/dextran for 40 h at 37°C. Tzm-bl cells were fixed in glutaraldehyde (0.2%) and stained with an X-gal substrate. HIV-infected cell spots were counted using an Immunospot Series Analyzer (Cellular Technology, Cleveland, USA). Average and standard error of five-time repeats of counts on ELISpot were plotted in histogram form. P-value analysis was used to evaluate the signficant change of the infectivity of C177A mutation vs. prototype BMJ4.

### Accession Numbers

Coordinates have been deposited in the Protein Data Bank (accession code 3VRL).

## Supporting Information

Figure S1 **The A10F9 monoclonal antibody displays higher binding affinity to BMJ4 p24.** (A) EC_50_ calculation of various p24 proteins binding with the A10F9 mAb. The OD_450 nm_ vs. A10F9 concentration ELISA curves were fitted to a half point of effective concentration (EC_50_), and the value is shown in brackets. (B) The binding affinity constant of the A10F9 monoclonal antibody with the BMJ4 p24 proteins was measured with a Biacore biosensor assay. (C) A10F9 binding with native HIV in immunofluorescence images. The DAPI-stained nucleus is shown in blue, and A10F9 mAbs recognizing pNL4-3 or BMJ4 p24 was visualized by a FITC-labeled goat anti-mouse antibody in green.
(TIF)

Figure S2 **Gel filtration purification of BMJ4 p24 protein in complex with A10F9 Fab.** The purification was performed in Agilent1200 HPLC system with G5000PW_XL_ column. The major peak demonstrating as bigger size with respect to the peak of excess A10F9 Fab was collected for concentrating and crystallization. Dark green, the analysis of individual BMJ4 p24; Cyan, the analysis of individual A10F9 Fab; Magenta, the resultant chromatography profile of the purification of the BMJ4-A10F9 complex.
(TIF)

Figure S3 **A10F9 monoclonal antibody displays binding ability to BMJ4 p24 CTD.** (A) The C-terminal domain (CTD) of BMJ4 p42 resolves as dimer in non-reducing condition but dissociates to monomer with DTT treat, which is consistent with the full-length BML4 p24 protein. (B) A10F9 monoclonal antibody displays binding ability to BMJ4_CTD_ instead of BMJ4_NTD_. 1, BMJ4_NTD_-reducing; 2, BMJ4_NTD_-nonreducing; 3, Protein MW Marker; 4, BMJ4_CTD_-reducing; 5, BMJ4_CTD_-nonreducing.
(TIF)

Figure S4 **A selected antibody panel against the HIV p24 capsid protein.** (A) Broad-spectrum anti-p24 antibodies were characterized by various p24 binding tests. (B) The anti-p24 antibodies were grouped and weighted by mutual cross-blocking rate evaluations.
(TIF)
