# Supplementary figures and images for: Structure of a Novel Shoulder-to-Shoulder p24 Dimer in Complex with the Broad-Spectrum Antibody A10F9 and Its Implication in Capsid Assembly
Source: PLoS One. 2013 Apr 19;8(4):e61314. doi: 10.1371/journal.pone.0061314 (PMC3631186; doi:10.1371/journal.pone.0061314)

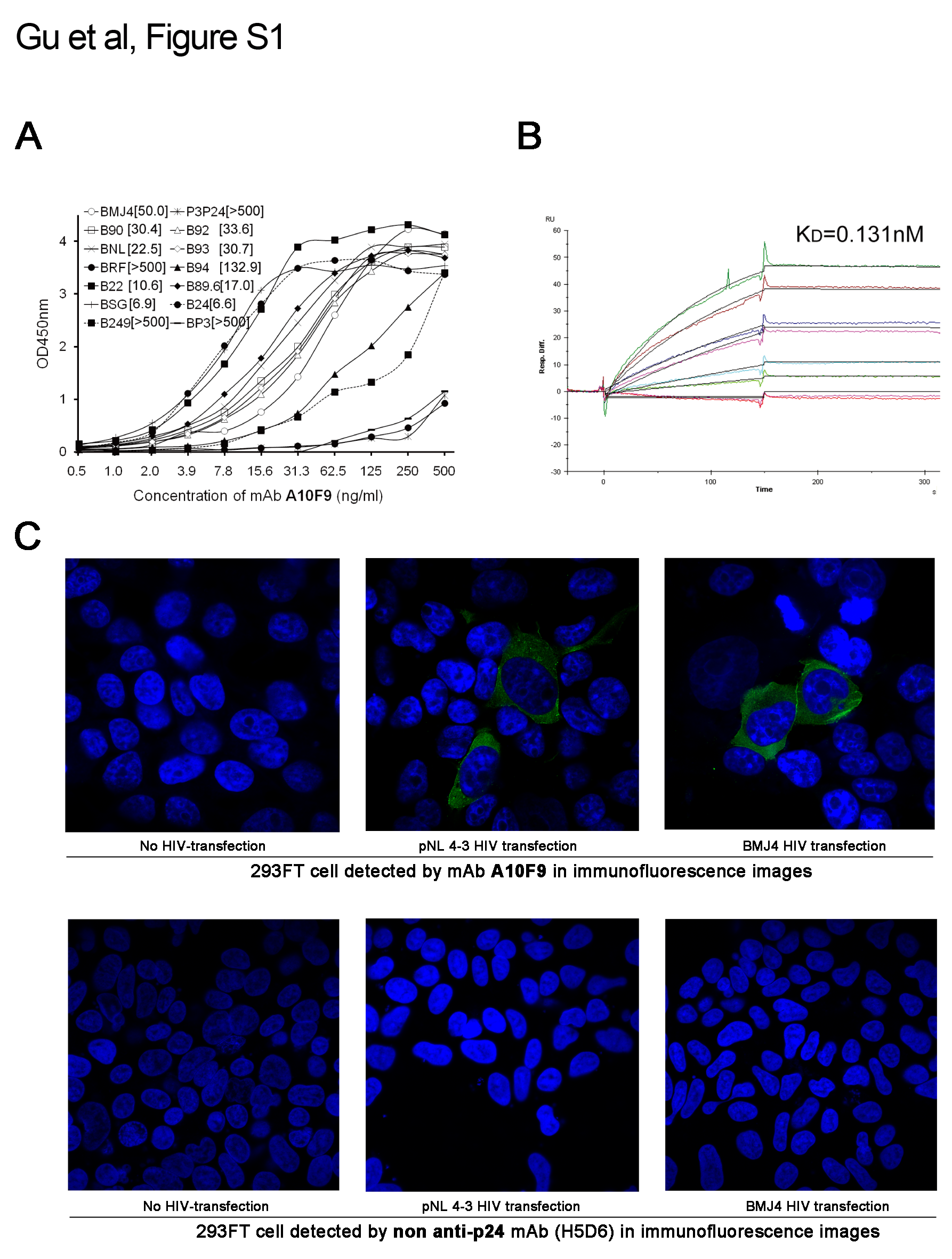

Supplement: Figure S1 — The A10F9 monoclonal antibody displays higher binding affinity to BMJ4 p24. (A) EC50 calculation of various p24 proteins binding with the A10F9 mAb. The OD450 nm vs. A10F9 concentration ELISA curves were fitted to a half point of effective concentration (EC50), and the value is shown in brackets. (B) The binding affinity constant of the A10F9 monoclonal antibody with the BMJ4 p24 proteins was measured with a Biacore biosensor assay. (C) A10F9 binding with native HIV in immunofluorescence images. The DAPI-stained nucleus is shown in blue, and A10F9 mAbs recognizing pNL4-3 or BMJ4 p24 was visualized by a FITC-labeled goat anti-mouse antibody in green. (TIF) [file pone.0061314.s001.tif]

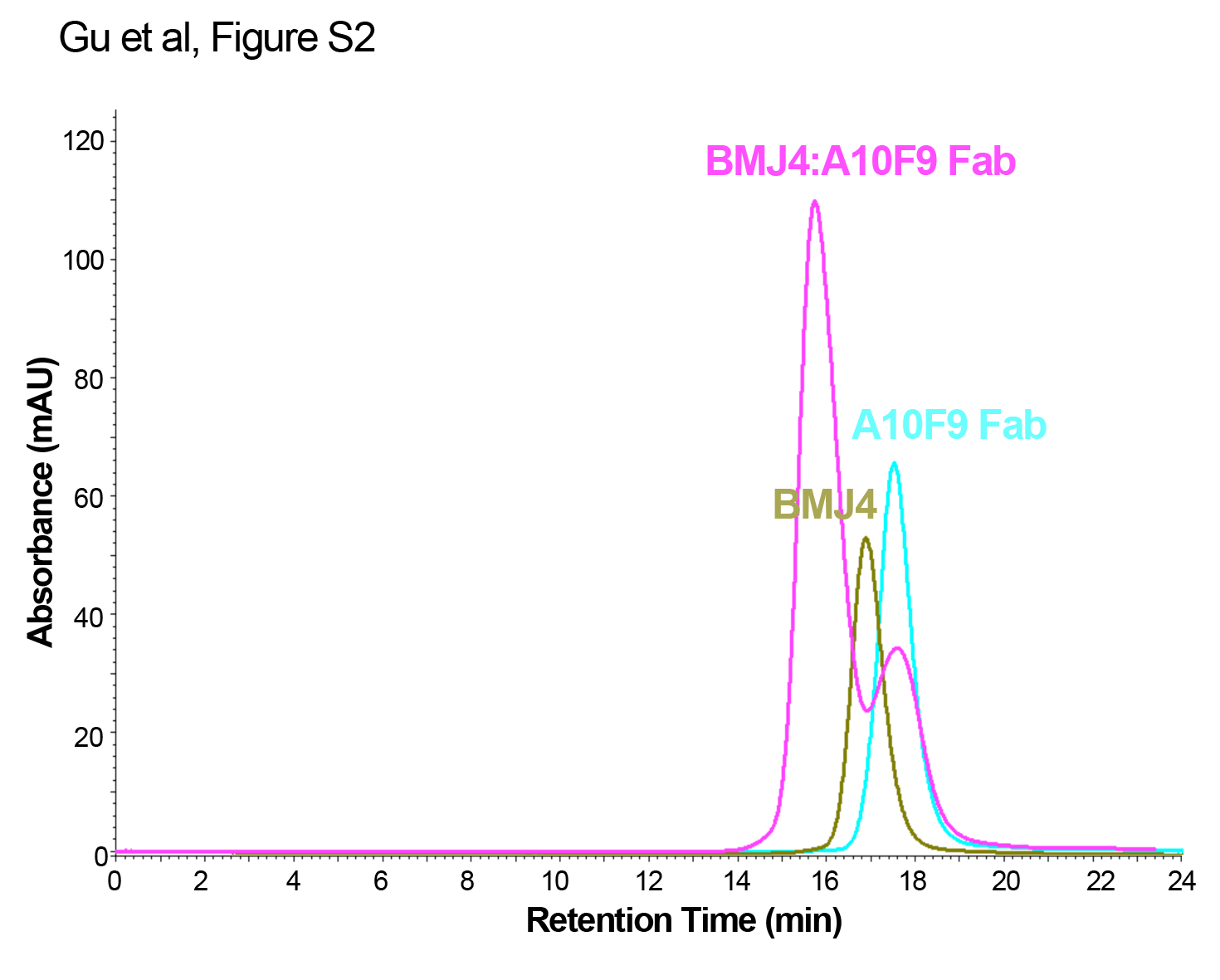

Supplement: Figure S2 — Gel filtration purification of BMJ4 p24 protein in complex with A10F9 Fab. The purification was performed in Agilent1200 HPLC system with G5000PWXL column. The major peak demonstrating as bigger size with respect to the peak of excess A10F9 Fab was collected for concentrating and crystallization. Dark green, the analysis of individual BMJ4 p24; Cyan, the analysis of individual A10F9 Fab; Magenta, the resultant chromatography profile of the purification of the BMJ4-A10F9 complex. (TIF) [file pone.0061314.s002.tif]

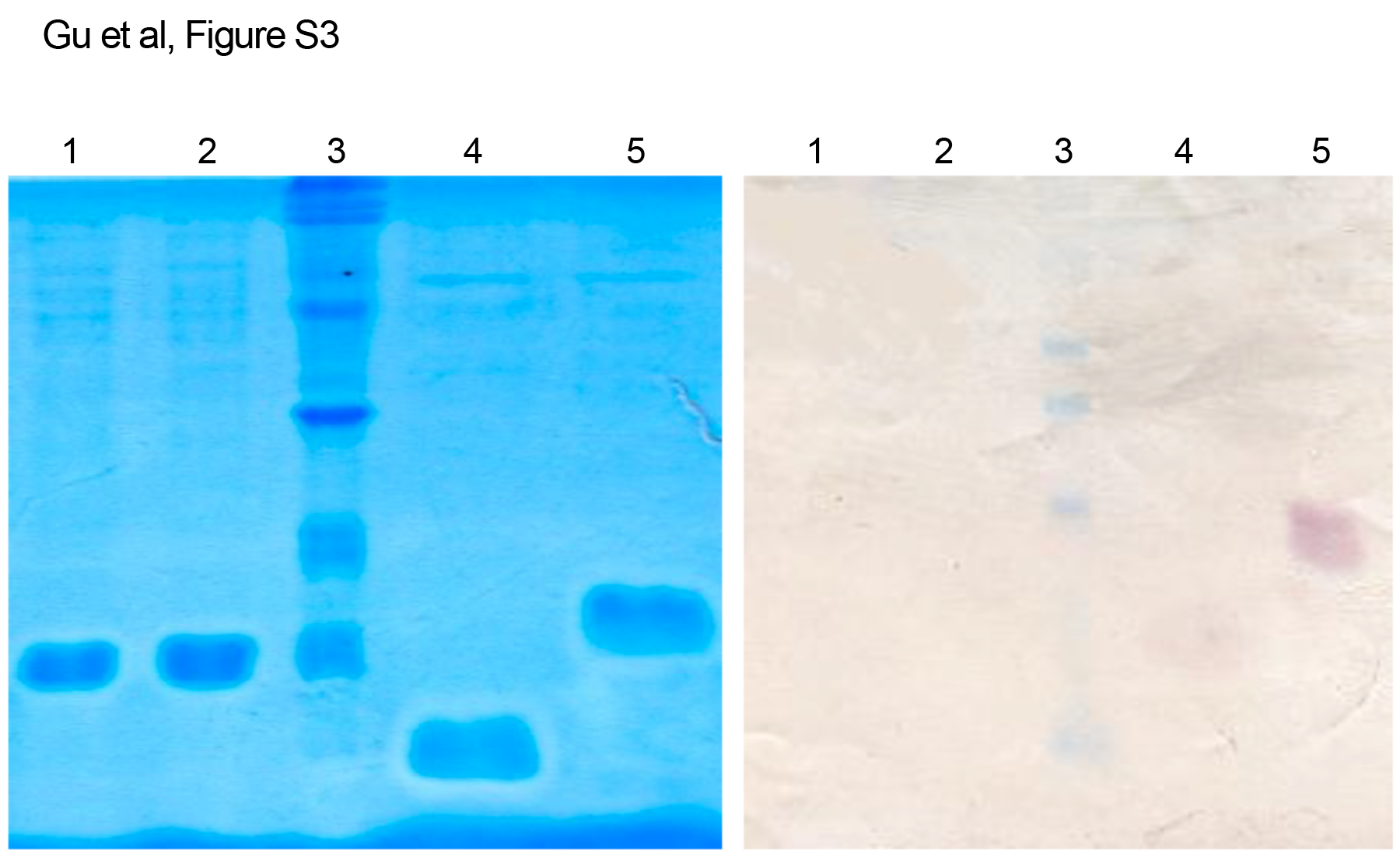

Supplement: Figure S3 — A10F9 monoclonal antibody displays binding ability to BMJ4 p24 CTD. (A) The C-terminal domain (CTD) of BMJ4 p42 resolves as dimer in non-reducing condition but dissociates to monomer with DTT treat, which is consistent with the full-length BML4 p24 protein. (B) A10F9 monoclonal antibody displays binding ability to BMJ4CTD instead of BMJ4NTD. 1, BMJ4NTD-reducing; 2, BMJ4NTD-nonreducing; 3, Protein MW Marker; 4, BMJ4CTD-reducing; 5, BMJ4CTD-nonreducing. (TIF) [file pone.0061314.s003.tif]

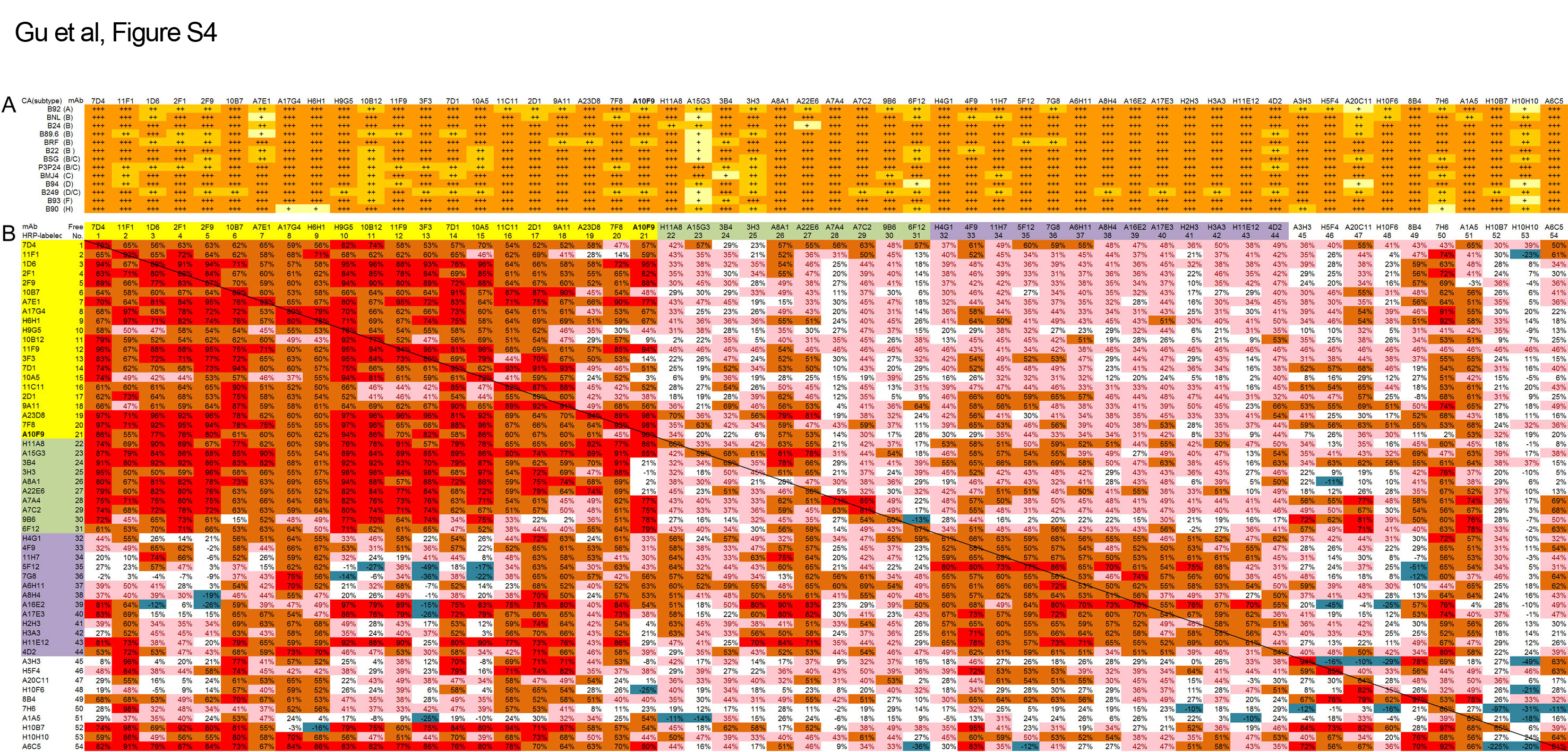

Supplement: Figure S4 — A selected antibody panel against the HIV p24 capsid protein. (A) Broad-spectrum anti-p24 antibodies were characterized by various p24 binding tests. (B) The anti-p24 antibodies were grouped and weighted by mutual cross-blocking rate evaluations. (TIF) [file pone.0061314.s004.tif]
